# Quantum Nanochemistry: 5-Volume Set. By Mihai V. Putz

**DOI:** 10.3390/ijms17081358

**Published:** 2016-08-19

**Authors:** Petr Čársky

**Affiliations:** J. Heyrovský Institute of Physical Chemistry, Academy of Sciences of the Czech Republic, Dolejškova 3, 18223 Prague 8, Czech Republic; petr.carsky@jh-inst.cas.cz

This book, with its 2889 pages in five volumes, represents an impressive piece of work written by a single author. The wide variety of topics covered by the book reveals the author’s deep and encyclopedic knowledge of quantum theory. The author identifies himself as a theoretical physicist, and characterizes his book as a compilation of his lecture notes which he has used within his classes. The author’s favorite topics can be easily recognized in all five volumes. The sections on the history of quantum theory examine concepts and fundamental quantum theories in retrospect, and may prove useful and amusing reading for lecturers teaching quantum theory. Moreover, the unconventional look at some problems related to quantum theory may prove inspiring for experts actively working in the field. Students may find some useful material in the book, although it seems more difficult to read than many other standard textbooks on quantum theory so far published. 

The title of the book may invoke an expectation that the content deals with theories and quantum chemical computational methods developed for the understanding of elementary steps in complex chemical processes occurring in modern nanotechnologies. However, this is not the case. The book does not deal with nanochemistry at all. Nor is it a textbook, for the most part. As stated in the preface, the book narrates the “story of quantum chemistry” as an extended review paper. This is unlike content currently available in textbooks, which are written as monographs displaying chapters as themes of interest. A somewhat disturbing feature of the book is the author’s overselling of his own research in some places. For instances, the author barely cites the work of established coryphaei in areas of quantum theory. The book may therefore be recommended to libraries of university departments of physics and physically oriented research laboratories, although less so to chemists interested in the theory of nanochemistry.

Volume I: (Quantum Theory and Observability) deals with the fundamental concepts of quantum theory. It contains five chapters.

Chapter 1 (Phenomenological Quantification of Matter, 63 pages) covers topics that are typical within introductory sections of textbooks on quantum theory. Special attention is paid to the explanation of unification of corpuscular and undulatory natures of matter.

Chapter 2 (Formalization of Quantum Mechanics, 96 pages) provides a survey of the main formal concepts of quantum theory that are used in the mathematical description of quantum phenomena treated in Chapter 1. The topics covered are concepts and tools for the mathematical description of the wave function, the correspondence principle, and the bra-ket formalism.

Chapter 3 (Postulates of Quantum Mechanics: Basic Applications, 192 pages). In this chapter, attention is paid mainly to quantum tunneling, the Schrödinger equation, Laguerre polynomials, vibrational motion, free electronic states in solids, quantum transition and scattering theory. Derivation of working formulas and equations are presented in a detailed and step-wise fashion, enabling easy understanding. However, to undertake a more in-depth study of vibrational states it would be more practical to use a textbook dedicated to vibrational spectroscopy. Additionally, the section on scattering theory does not bring anything new to the table in terms of nanochemistry. It contains only textbook application of the Yukawa potential and derivation of the Rutherford scattering equation. 

Chapter 4 (Quantum Mechanics for Quantum Chemistry, 190 pages). This chapter can be viewed as an extension of a series of other previously published textbooks on quantum chemistry. A reader who is interested in quantum theory relevant for nanochemistry may find its contents unbalanced. Some sections are too specialized, such as those dealing with path integrals; whilst others are too simple, such as those dealing with electronegativities and chemical hardness that are not so important for modern nanochemistry. Hartree–Fock and density functional theory are described in detail, but advanced computational methods are noted only by a few sentences. 

Volume II: Quantum Atoms and Periodicity. It contains five chapters.

Chapter 1 (Historical Highlights on the Periodicity of the Chemical Elements, 62 pages). This chapter describes, in a narrative way, the evolution of scientific knowledge from the ancient Greeks to Mendeleev. It is enjoyable to read, however it does not fit in the scope of the book.

The following chapters present abrupt change—they present unconventional view on the electronic structure of atoms, with plenty of equations and concepts, making the text difficult to read. Rigorous derivations are presented with the aim of discussing fuzzy chemical concepts, such as electronegativity and chemical hardness.

Chapter 2 (Quantum Assessment for Atomic Stability, 42 pages). The two main topics of this chapter are periodic path integrals and the Feynman–Kleinert variational formalism. This chapter is purely theoretical and serves as a preparation for the next section.

Chapter 3 (Periodicity by Quantum Propagators in Physical Atom, 55 pages). The path integral approach is used in this chapter to provide a new definition of electronegativity and chemical hardness. It is shown that this new approach can eliminate irregularities in periodicity often found in traditional models. 

Chapter 4 (Periodicity by Peripheral Electrons and Density in Chemical Atoms, 199 pages). As in other places in this book, electronegativity and hardness are taken as the major electronic indicators of structure and reactivity. They are expressed in different ways and discussed in connection with valence atomic structure, density functionals, electrophilicity, diamagnetic susceptibility, polarizability and ionization potentials. This was undertaken with the aim of showing the periodicity of these atomic properties. The conclusion of this chapter is certainly disappointing for quantum chemists. It claims boldly that this enterprise was undertaken for the future understanding of chemical bonding, reactivity, aromaticity, all the way up to the modelling of biological activity.

Chapter 5 (Quantum Algebraic and Stochastic Dynamics for Atomic Systems, 130 pages). This chapter deals with isolated atoms—the term “atomic system” refers to a single atom, not a system of atoms. It deals with two unconventional methods for describing electron interactions in atoms. This is achieved by abstract formalization within the quantum algebra of open systems and by an analytical formulation within stochastic/dissipative systems. Although it may present a novel approach, it is not clear how this methodology could lead to the expected outcome of a better understanding of the binding in molecules and chemical reactivity. 

Volume III: Quantum Molecules and Reactivity. It contains four chapters.

Chapter 1 (Modern Quantum Nature of the Chemical Bond: Valence, Orbitals and Bondons, 91 pages). This chapter represents the author’s original perspective on the nature of the chemical bond. Instead of electronic interactions, a chemical bond is assumed to be formed by a virtual chemical field and its corresponding quasi-particles, called bondons. The chemical field is characterized by a bond length and a bond energy. As a consequence, bondons have a strange features, having differing mass and charge for each chemical bond. The treatment of bondons in this chapter is an interesting exercise in theoretical physics, but it is however difficult to conceive how the concept may become a practical modern theory of chemical bonding. Particularly, when the main focus of this chapter is an application of bondons theory to Bose–Einstein condensation. Bondons are noted also at other places in the book, but again with not much convincing evidence with respect to their utility.

Chapter 2 (Molecular Structure by Quantum Chemistry, 131 pages). This chapter begins with an incomprehensible abstract containing a single sentence which straddles over on 15 lines. The formula for the energy of harmonic oscillator is then derived and an analysis of bonding in the van der Waals molecule He_2_ is selected for its useful application. Following this derivation, sections dealing with the localization of orbitals and the fundamentals of group theory are encountered. Their use for the formation of symmetry adapted orbitals and symmetry in the crystal field theory are well described. However, much of the content may be readily found in standard textbooks on quantum chemistry.

Chapter 3 (Quantum Chemical Reactivity of Atoms-In-Molecules, 215 pages). Once a promising computational method, Atoms-In-Molecules (AIM) is nowadays somewhat of an obsolete topic. This chapter advocates its revival by means of combination of electronegativity and hardness. As claimed in the abstract, such an approach can generate a plethora of density functionals for use in quantifying the “many-electronic” structures and their transformation at the conceptual rather than at the computational quantum level of comprehension. Throughout the book, the conceptual aspect is strongly favored. However, the utility of AIM in its new form is not documented enough. Rich tabular material on electronegativity and hardness, obtained in different ways, is limited to atoms and small molecules that can be treated by rigorous quantum chemical methods. Additionally, their electronic structure and reactivity can be better explained in terms familiar to chemists. Anthocyanidins is the only class of larger molecules treated in this chapter, unfortunately, without any clear validation of the utility of an AIM approach.

Chapter 4 (Modeling Molecular Aromaticity, 81 pages). This chapter deals with the problem of aromaticity by means of hardness and AIM, i.e., by tools described in previous chapters. 

Volume IV (Quantum Solids and Orderability) contains 5 chapters.

Chapter 1 (Bondons on Graphenic Nanoribbons with Topological Defects, 73 pages). This chapter addresses the topical problem of the formation of topological defects, isomerism and phase transitions in 2D monolayers such as graphene. It presents a detailed theoretical study on the evolution of a nanoribbon defect structures, based on the path integral formalism and bonding by bondons. The description of the model in Sections 1.3.1 to 1.3.3 is difficult to read, and general comments in the “Conclusion” does permit the reader to make a proper assessment of the utility of this new approach. In any case, one is in agreement with the author that, a “further works are needed for validation of the model”. 

Chapter 2 (Geometrical Crystallography, 173 pages). This chapter is more conventional and it presents an elementary introduction to crystallography by description of fundamental concepts such as geometry of crystallographic cells, crystallographic system, and point and space groups. All this can be found in commonly available literature. This chapter does not belong in a book on quantum nanochemistry. This chapter could be usefully skipped and the volume could be easily continued by the next chapter that follows. 

Chapter 3 (Quantum Roots of Crystals and Solids, 93 pages) is an elementary introduction to the theory of solid states. The chapter is a useful survey of quantum mechanics relevant to solids, although a more readable survey could be found in Kittel’s “Introduction to Solid State Physics” (as quoted in “Specific References”). 

Chapter 4 (Chemical Crystallography, 137 pages). This chapter is again a compilation of commonly available literature.

Chapter 5 (X-ray Crystallography, 132 pages). This chapter is a comprehensive description of the underlying theory of this technique. 

Volume V (Quantum Structure—Activity Relationships (Qu-SAR)).

In this volume, the author explores how to give a quantum description to more or less empirical QSAR approaches which, by means of orthogonal correlations, should lead to a unified SAR theory of chemical-biological interactions. This volume could not be classified as a textbook because it uses many terms and concepts that are not satisfactorily explained, making the reading of the volume difficult to understand for non-experts. The volume may be more usefully characterized as the author’s personal take on problems commonly encountered in the field of structure–activity relationships. The volume is divided into three chapters. 

Chapter 1 (Logistic Enzyme Kinetics, 77 pages) starts with the Michaelis–Menton equation and continues with its later variants, thus providing a theoretical background to enzyme kinetics.

Chapter 2 (Statistical Space for Multivariate Correlations, 114 pages) presents the derivation of equations used for multiparametric correlations. The aim of this chapter is to make the reader familiar with mathematical tools previously published in special monographs and to prepare the reader for more advanced types of QSAR which is treated in the next chapter. 

Chapter 3 (Chemical Orthogonal Spaces for Structure-Activity Relationship (COS-SAR), 375 pages). This chapter presents the author’s original and fresh ideas about general and quantitative structure-activity relationships. The general theory is called Quantum-SAR. Its key feature is the transformation of structured data to an orthogonal basis with the aim of eliminating mutual dependence of the structural descriptors. However, the derived equations become exceedingly complex and difficult to understand. It is likely that, because of its complexity, the description loses its chemical and pharmacological context. Instead of general comments in “Conclusions” it would be more beneficial to present a practical assessment of the rich tabular material.

